# Supplementary material for: Axl Expression in Renal Mesangial Cells Is Regulated by Sp1, Ap1, MZF1, and Ep300, and the IL-6/miR-34a Pathway
Source: Cells. 2022 Jun 9;11(12):1869. doi: 10.3390/cells11121869 (PMC9221537; doi:10.3390/cells11121869)
Supplement: Supplementary file 1 [file cells-11-01869-s001.zip › cells-1737646-supplementary.pdf]

Supplementary File S1. Sequencing Results for pGL4-Axl-4.4-luc2 Plasmids. The pGL4.0 plasmid containing Axl-UTR promoter region was sent to the CD Genomics for sequencing. Results were reported by the company.

Sequence No. 1

>412_pGL4_Axl_4_4_luc2_41_2_NA10_CIRCLE_8683_l1

gttggactcaagacgatagttaccggataaggcgcagcggtcgggctgaacg

GGGGGTTCGTGCACACAGCCCAGCTTGGAGCGAACGACCTACACCGAACTGAGATACCTACAGCGTGAGCTATGA
GAAAGCGCCACGCTTCCCGAAGGGAGAAAGGCGGACAGGTATCCGGTAAGCGGCAGGGTCGGAACAGGAGAGC
GCACGAGGGAGCTTCCAGGGGGAAACGCCTGGTATCTTTATAGTCCTGTCGGGTTTCGCCACCTCTGACTTGAGC
GTCGATTTTTGTGATGCTCGTCAGGGGGGCGGAGCCTATGGAAAAACGCCAGCAACGCGGCCTTTTTACGGTTCC
TGGCCTTTTGCTGGCCTTTTGCTCACATGTTCTTTCCTGCGTTATCCCCTGATTCTGTGGATAACCGTATTACCGCCTT
TGAGTGAGCTGATACCGCTCGCCGCAGCCGAACGACCGAGCGCAGCGAGTCAGTGAGCGAGGAAGCGGAAGAG
CGCTGCCGGCACCTGTCCTACGAGTTGCATGATAAAGAAGACAGTCATAAGTGCGGCGACGATAGTCATGCCCCG
CGCCCACCGGAAGGAGCTGACTGGGTTGAAGGCTCTCAAGGGCATCGGTCGACGGATCCTTATCGATTTTACCAC
ATTTGTAGAGGTTTTACTTGCTTTAAAAAACCTCCCACACCTCCCCCTGAACCTGAAACATAAAATGAATGCAATTG
TTGTTGTTAACTTGTTTATTGCAGCTTATAATGGTTACAAATAAAGCAATAGCATCACAAATTTCACAAATAAAGCA
TTTTTTTCACTGCATTCTAGTTGTGGTTTGTCCAAACTCATCAATGTATCTTATCATGTCTGCTCGAAGCGGCCGGCC
GCCCCGACTCTAGAATTATTACACGGCGATCTTGCCGCCCTTCTTGGCCTTAATGAGAATCTCGCGGATCTTGCGG
GCGTCCAACTTGCCGGTCAGTCCTTTAGGCACCTCGTCCACGAACACAACACCACCGCGCAGCTTCTTGGCGGTTG
TAACCTGGCTGGCCACATAGTCCACGATCTCCTTCTCGGTCATGGTTTTACCGTGTTCCAGCACGACGACTGCGGC
GGGCAGCTCGCCGGCATCGTCGTCGGGCAGGCCGGCGACCCCGGCGTCGAAGATGTTGGGGTGTTGCAGCAGGA
TGCTCTCCAGTTCGGCTGGGGCTACCTGGTAGCCCTTGTATTTGATCAGGCTCTTCAGCCGGTCCACGATGAAGAA
GTGCTCGTCCTCGTCCCAGTAGGCGATGTCGCCGCTGTGCAGCCAGCCGTCCTTGTCGATGAGAGCGTTTGTAGCC
TCGGGGTTGTTAACGTAGCCGCTCATGATCATGGGGCCACGGACGCACAGCTCGCCGCGCTGGTTCACACCCAGT
GTCTTACCGGTGTCCAAGTCCACCACCTTAGCCTCGAAGAAGGGCACCACCTTGCCTACTGCGCCAGGCTTGTCGT
CCCCTTCGGGGGTGATCAGAATGGCGCTGGTTGTTTCTGTCAGGCCGTAGCCCTGGCGGATGCCTGGTAGGTGGA
AGCGTTTGGCCACGGCCTCACCTACCTCCTTGCTGAGCGGCGCCCCGCCGCTGGCGATCTCGTGCAAGTTGCTTAG
GTCGTACTTGTCGATGAGAGTGCTCTTAGCGAAGAAGCTAAATAGTGTGGGCACCAGCAGGGCAGATTGAATCTT
ATAGTCTTGCAAGCTGCGCAAGAATAGCTCCTCCTCGAAGCGGTACATGAGCACGACCCGAAAGCCGCAGATCAA
GTAGCCCAGCGTGGTGAACATGCCGAAGCCGTGGTGAAATGGCACCACGCTGAGGATAGCGGTGTCGGGGATGA
TCTGGTTGCCGAAGATGGGGTCGCGGGCATGACTGAATCGGACACAAGCGGTGCGGTGCGGTAGGGCTACGCCC
TTGGGCAATCCGGTACTGCCACTACTGTTCATGATCAGGGCGATGGTTTTGTCCCGGTCGAAGCTCTCGGGCACGA
AGTCGTACTCGTTGAAGCCGGGTGGCAAATGGGAAGTCACGAAGGTGTACATGCTTTGGAAGCCCTGGTAGTCG
GTCTTGCTATCCATGATGATGATCTTTTGTATGATCGGTAGCTTCTTTTGCACGTTGAGGATCTTTTGCAGCCCTTTC
TTGCTCACGAATACGACGGTGGGCTGGCTGATGCCCATGCTGTTCAGCAGCTCGCGCTCGTTGTAGATGTCGTTAG
CTGGGGCCACAGCCACACCGATGAACAGGGCACCCAACACGGGCATGAAGAACTGCAAGCTATTCTCGCTGCACA
CCACGATCCGATGGTTTGTATTCAGCCCATAGCGCTTCATAGCTTCTGCCAGCCGAACGCTCATCTCGAAGTACTCG
GCGTAGGTAATGTCCACCTCGATATGTGCGTCGGTAAAGGCGATGGTGCCGGGCACCAGGGCGTAGCGCTTCATG

GCTTTGTGCAGCTGCTCGCCGGCGGTCCCGTCTTCGAGTGGGTAGAATGGCGCTGGGCCCTTCTTAATGTTTTTGG
CATCTTCCATGGTGGCTTTACCAACAGTACCGGATTGCCAAGCTTGGCCGCCGAGGCCAGATCCTCTAGAGTCGAG
GAATTCGAAGTTGATGGGCACCTCGCTAGGAAAGGGGGCAGGGCCGGGTTGTCCCCGGCCCTCCCCCCACCCCTG
GGCTCCTGGGATTTGGCACTGCCCGCCTGGCACAGCGGGAGCCCCTCGGCTGGCTCAACTCCTCCGCAGCTAGCC
GCCTTCCCGGCTACCCTGCCTCATTCTCTCACTCCCAGACTTCTTTCCCCGCCCCTGGCTCCCCCTCTGCCTCCGCCCA
CTAGGGCCCCAGCCTTGGGGCCACTGGGACTTGGAGGGGTTAACTAGGAGTGAAGAGTACGGGATCTGCCTTGT
CTTAAAGGAGCCTGGGCGCCTTGGAGAGAGGCTGGGGAGGAGGAAGGAGACCTTGGTGGCTCATGGTGGCTGA
GGCCGATCCTGGGCTGGTGAGTTAGGAGGATGGGCGCTGTGTCCCCCTGAACTCTCAGGGTCTGGTAAACATTCC
TGTAGAATTTCCACTCCTCTCAGCCCAAGACGCCCCCCAGCCCCCCTTTCTGGGCCCCCACCCTCAGCCCAGCCTGC
CAGGCCTCCTCCAATTCCCCTCACACTCGCAGCTTGAACAAGGACTCTCTCTCTCTCTCTCTCTCTCTCTCTCTCTC
TCTCTCTCTCTCTCTCTCTCTCTGCATTGACACAGAAGCTGGGACAGGTAGAGACATGCAATGGCATGCTGCCTGCC
CACAGATACCCTCAGATGAGCCGGGTCAGGAGCTTGCCTGGGGAGAAGTCTTACAAATGGAAGGTCACTGCGGG
ACTGGCATATGGGGGATCAGGGTACTCACTGAGGGCACATCAACACTCAAACCCACCGAAGCCCACTTGAACACA
GAAACAGGAATCCAGAGGTGGCAGCAGACTGAAGTGACAACCCCAGCTCTAGCCCAGCCACATGACTTTGGGCA
ATGGGCAAATGCCTGTCTTCCTGTACCTCAGTGTGTCTAGCTTTAAGATGGGGACGAGGACAGCCCCTGCTAATGC
AGCAGGCTAGTGCGAGAATGATCTAGGTCAAAGTGCATGCAGTTTCTTGCACAGGGTGGCCCAGATGACGAGGT
GCCCGGGATTTTTGCTACCCATGCTGGTTGTTCCTCTGCCTCAGGATTCTTGTATCCAGCCCTGGCAGATCCGGAGC
CAGTATTAAGCCTCTGCTGCATGTCTGCTTCTATGCCTCTAGGTGTTTGCCATAGAGGGCATGCTCAGATGGCCTCC
TAGTACCCAGTTCTCTTCTTCATTAGTCAGGCTGCTTCTTGATCAAATCATTGAGCTGCTTTTGATCACCACAGAATC
CCATGTCACCTCCTGGCATCTCTCTCTCTCTCTCTCCCTTTGCCCTTCTCCATATCCACCACTCCTCCTTCCATCCCCCT
GCCTCCAATCCATCTCTTGGTTGGGATCTAGGAGGATGTTTCTGAGACAGCCCTCCCCCCCATGTCTGTCCTCTAGA
CTCCCCACTTCTCTGATGATCAACTCCAAGCCTCTCAGCTGCTCGCTCTCGGTCCTAACCATAACCACCATCCTGTTT
GTATCTTGATTCAGTGATGTGGCAGCCCCGGCATCTAACCTGCCACCTAGTCTGAGCCCTGGGGTCCTCCTGAACG
CTCAGTGTGGATTGGTTGCATGGAATCCCAGGGAGAGCTCAGCCTTACAAACCTCTCTCCTATGTGGCTGCCTCTTT
AGAGTCTCACCCTGGTCTAGCCTTGCCCTTTCCCTCCTGGATCCCAGCTATAATTTGCTCCTTCAGTGGCCCGATTGC
TTCTTCCATCACGTTTCCCTTTTAGCAGCCACAGGGTGTTTCTTATCCCACATCCCATCTGATCCTTTCCTTCTCCCTTC
CTTCCCTCTCCGATACCACCTAGCTGTATCCCATCTTCTGCTTCAGCCTTTAGGATAAAGCCACGGGCTTCTTACAAG
CACCCCTCATTGCCACCTGAGCGTGTCCTCACCTCCCTGGTCAGTTTTCATTTTCTCACCCGAAACAGCTCCCTCACA
GGCTGTGGAACAGCTTTCTGTGGCACTTTGTCCCAAGCTCCTACACCTCCCTCAGCCCCACCTCTCCTCCCTTTTCTT
TTGCATCCTTTCCAGCCCCCTCCTGTCTAACCTAGCTCTACTCCTTCAATATACGGCTCCCTCTCAGCACCGCCTTTTT
CTGAGATGGTCTCACTATATCACCAAGGGTGGTCTTGAACTCTCAGTCCTACGTGCTTTGCCCTCCAGAGAGTGCT
GGGTGACAGGCAAGCACCACCACACCCAGCTTACCCGGTGCTTTTCATTGGCAGCAGGCCCCGTTTATTGTCATTA
TGGAGTTATTGCACAATTCTGTTTCCTGCCTGTCTCTCCTTTCTGCTGGTGGACGCTGGGAGGACAGCCTGGGGGA
TATCGCAGATATTGCTGCAACCCTTAGAAAAGGCCTTGCCATACTGTAGATAAGAAGAAAAAAAGGCATCTCTGG
GGTGAAGTGGAAACTTTAGGGTTCTTTGGAAAGTCGGTTGGATGATATTTTGCTGGGGCAAACACATGAAGGGAC
GTTTCATTAAAGCAGACATAGGTGTGAAAGGCTAAGGTAGACTCATGAAGGAATGTTTCACTGAGAGACACACAC
AGGAGAGAGGATGTTCTGCTAAAGCAAGTGTGTGAAGGGACACGTGATGAAGGATTCTTTGCTAACAGCACTCA
GGTATTGGTCTGCCTTACGTTGCATAGTTGATCTGCTTTTGTTGGGACTCCACAGACTTGGGCTGGCTGCGGCAGA
CTTGGGTGGAGGATGCATGATGTTTGGTGGGTGTTGGAGATCGGGCCTAATGCTTTGATTTGACAGGGAATGTGC
TTGTTTGTGCAAACACTAAAGTTAGTTTTTGATTGATCAATAAAGATGCCAGTGACTAATGGCTGGGCAAAGGGAG
GCAGGGTGGGACCCTTAGAGTTGGTGGGGCTAGGACACAGGGGAAAGGAGGAAACGCAGACTTTCAGGATCAC
TGTGAATCACGGGGTTGGGGGGAGCGTGGAGAGAGATGGATCAGATTTAGAGCTGCAGAGGGAACATCATCTG
AAATGTAGGGAAAGCGGCCGCCGGAAAGGCTGCTCAGAAACACCTTGGGCATCAGAGACCAATGGGCCCTACAG
AAGGTAACAGCGCAGCAAAGTTAAAAGTAGATCCAAGGAGGCTGGAGAGGTGGCTCAGCAGTTAAGAGCACTG

GCTGCTCTTCCAGAGGTCCTGAGTTCAATTCCCAGCAACCACATGGTGGCTCACAACCATCTGTAATGGGATCTGG
CGTGTCTGAAGACAGCTACAGTGCACTTACATACATTAAATAAATAAAATCTTTTTAAGAATGCAAAATATGTATT
TAATGTTTCTAACCAGTACAAAGACCAAAGGCCACATTTACTCTAGGTTAGTCACAAACCAATTATTTAATAGAAGC
TATAAAATAATTGGGAAGAATATTTGGAATATCTCAAAAGATTTAAAATAGGAGGACACTTTACAAAAATCCGCTT
AGGAAATAAAATTCCTCACCTGTATGTAGAAATGTAACATTGCAGGGGAGCTTATAAAATACAAAGACAAGTATA
AAAGGACTGTGTACTCTAAATCCAAAAACTCAATAATAACCAAATACTTAGTTTTAAAATTTACAGTTCCTGGGTAT
GGACCACATTATTATACACATCTTCCAAGACAGCAATCATTATTCTTCAGTTAACCATGTGAGGTATCGTCTTCTACA
AAATCTTTGGCCATCTCTGCTGTGATCACATCAATGTGACTAACCTTATTTCTGGACTTTACCCCATAGAATTTGTCA
GCTGACTCAAGCAGTTCAGGCCTAAAGGCAGTAGTAATAAACTGGGCATGCACAGCTAGCGCTATAATCATATCT
GACACAGCTTTTCTGTGTTGAGCATCCAAAGCTTGGTCGATCTCATCAAACAGGTAAAAAGGAGCGGGGTCACATT
TCTGAATGGCAAAAATGAGAGCAAGGGCTGCCAGAGATTTCTGTCCTCCTTAAAGCTGTTGCATTTCTCTCAACTCC
CCTTGCTTCCCTGTAAACGACACCCTGACCCCAACTCGTGTGGACTGGTCAACTGTTGGAACACTGCTCTGTGACCC
AGAGCCCCGTTCGCTCTCGCCGCTCCCTTCTCCTTCGTCCTGAGACTGGCTGCCCTCCACATTTCCTTTCTTCATCAC
CAAAGTTGCTTTGCCACCGGGTACCAACTTCTGAAAAACTTCACTGAAGTTCTTAGATACCTGTTTGAAAGTTAACT
GAATAGCTTCATATTTTCTAAGTTCGAGTACATTCATCAATTCCATGATTGAAAGTCTTTTTTTAAAGTAGATTTAG
AAAGTGTTGAGCCAGGAGTACATGAGGGGAAAGCATGCTCGAGGCTAGCGAGCTCAGGTACCGGCCAGTTAGGC
CAGAGAAATGTTCTGGCACCTGCACTTGCACTGGGGACAGCCTATTTTGCTAGTTTGTTTTGTTTCGTTTTGTTTTG
ATGGAGAGCGTATGTTAGTACTATCGATTCACACAAAAAACCAACACACAGATGTAATGAAAATAAAGATATTTTA
TTGCGGCCTGTCCAATACCTCCCGTACCTTAATATTACTTACTTATCCTTGAGAGACGTACTAGTAACCCTGATAAAT
GCTTCAATAATATTGAAAAAGGACGAGTATGAGCATCCAACATTTTCGTGTCGCACTCATTCCCTTCTTTGCGGCAT
TTTGCTTGCCTGTTTTTGCACACCCCGAAACGCTGGTGAAAGTAAAAGATGCTGAAGATCAACTGGGTGCAAGAGT
GGGCTATATCGAACTGGATCTCAATAGCGGCAAGATCCTTGAGTCTTTCCGCCCCGAAGAACGATTCCCGATGATG
AGCACTTTCAAAGTACTGCTATGTGGCGCGGTGTTGTCCCGTATAGACGCCGGGCAAGAGCAGCTTGGTCGCCGT
ATACACTACTCACAAAACGACTTGGTTGAGTACTCGCCGGTCACGGAAAAGCATCTTACGGATGGCATGACGGTA
AGAGAATTGTGTAGTGCTGCCATTACCATGAGCGACAACACCGCGGCCAACTTACTTCTGACAACGATCGGAGGC
CCTAAGGAGCTGACTGCATTTCTTCATAATATGGGTGATCATGTGACCCGGCTTGACCGCTGGGAACCAGAGTTGA
ACGAAGCCATACCGAACGACGAGCGTGATACCACGATGCCAGTAGCAATGGCCACAACTCTTCGGAAACTACTCA
CTGGCGAACTTCTTACTCTAGCATCACGACAGCAGCTCATAGACTGGATGGAGGCGGACAAAGTAGCAGGACCAC
TTCTTCGCTCGGCCCTCCCTGCTGGCTGGTTCATTGCTGACAAATCGGGGGCCGGTGAACGCGGCTCTCGCGGCAT
CATTGCTGCGCTGGGGCCTGATGGTAAGCCCTCACGAATCGTAGTGATCTACACGACGGGGAGTCAGGCCACTAT
GGACGAACGAAATAGGCAGATCGCTGAGATCGGTGCCTCACTGATCAAGCACTGGTAACCACTGCAGTGGTTTAG
CATTTGCGGCCGCTGTCAGACCAAGTTTACTCATATATACTTTAGATTGATTTAAAACTTCATTTTTAATTTAAAAGG
ATCTAGGTGAAGATCCTTTTTGATAATCTCATGACCAAAATCCCTTAACGTGAGTTTTCGTTCCACTGAGCGTCAGA
CCCCGTAGAAAAGATCAAAGGATCTTCTTGAGATCCTTTTTTTCTGCGCGTAATCTGCTGCTTGCAAACAAAAAAAC
CACCGCTACCAGCGGTGGTTTGTTTGCCGGATCAAGAGCTACCAACTCTTTTTCCGAAGGTAACTGGCTTCAGCAG
AGCGCAGATACCAAATACTGTTCTTCTAGTGTAGCCGTAGTTAGGCCACCACTTCAAGAACTCTGTAGCACCGCCT
ACATACCTCGCTCTGCTAATCCTGTTACCAGTGGCTGCTGCCAGTGGCGATAAGTCGTGTCTTACCGG

Sequence No. 2

>4314_pGL4_Axl_4_4_luc2_43_14_NA11_CIRCLE_8685_l1

gttggactcaagacgatagttaccggataaggcgcagcggtcgggctgaacg

GGGGGTTCGTGCACACAGCCCAGCTTGGAGCGAACGACCTACACCGAACTGAGATACCTACAGCGTGAGCTATGA
GAAAGCGCCACGCTTCCCGAAGGGAGAAAGGCGGACAGGTATCCGGTAAGCGGCAGGGTCGGAACAGGAGAGC

GCACGAGGGAGCTTCCAGGGGGAAACGCCTGGTATCTTTATAGTCCTGTCGGGTTTCGCCACCTCTGACTTGAGC
GTCGATTTTTGTGATGCTCGTCAGGGGGGCGGAGCCTATGGAAAAACGCCAGCAACGCGGCCTTTTTACGGTTCC
TGGCCTTTTGCTGGCCTTTTGCTCACATGTTCTTTCCTGCGTTATCCCCTGATTCTGTGGATAACCGTATTACCGCCTT
TGAGTGAGCTGATACCGCTCGCCGCAGCCGAACGACCGAGCGCAGCGAGTCAGTGAGCGAGGAAGCGGAAGAG
CGCTGCCGGCACCTGTCCTACGAGTTGCATGATAAAGAAGACAGTCATAAGTGCGGCGACGATAGTCATGCCCCG
CGCCCACCGGAAGGAGCTGACTGGGTTGAAGGCTCTCAAGGGCATCGGTCGACGGATCCTTATCGATTTTACCAC
ATTTGTAGAGGTTTTACTTGCTTTAAAAAACCTCCCACACCTCCCCCTGAACCTGAAACATAAAATGAATGCAATTG
TTGTTGTTAACTTGTTTATTGCAGCTTATAATGGTTACAAATAAAGCAATAGCATCACAAATTTCACAAATAAAGCA
TTTTTTTCACTGCATTCTAGTTGTGGTTTGTCCAAACTCATCAATGTATCTTATCATGTCTGCTCGAAGCGGCCGGCC
GCCCCGACTCTAGAATTATTACACGGCGATCTTGCCGCCCTTCTTGGCCTTAATGAGAATCTCGCGGATCTTGCGG
GCGTCCAACTTGCCGGTCAGTCCTTTAGGCACCTCGTCCACGAACACAACACCACCGCGCAGCTTCTTGGCGGTTG
TAACCTGGCTGGCCACATAGTCCACGATCTCCTTCTCGGTCATGGTTTTACCGTGTTCCAGCACGACGACTGCGGC
GGGCAGCTCGCCGGCATCGTCGTCGGGCAGGCCGGCGACCCCGGCGTCGAAGATGTTGGGGTGTTGCAGCAGGA
TGCTCTCCAGTTCGGCTGGGGCTACCTGGTAGCCCTTGTATTTGATCAGGCTCTTCAGCCGGTCCACGATGAAGAA
GTGCTCGTCCTCGTCCCAGTAGGCGATGTCGCCGCTGTGCAGCCAGCCGTCCTTGTCGATGAGAGCGTTTGTAGCC
TCGGGGTTGTTAACGTAGCCGCTCATGATCATGGGGCCACGGACGCACAGCTCGCCGCGCTGGTTCACACCCAGT
GTCTTACCGGTGTCCAAGTCCACCACCTTAGCCTCGAAGAAGGGCACCACCTTGCCTACTGCGCCAGGCTTGTCGT
CCCCTTCGGGGGTGATCAGAATGGCGCTGGTTGTTTCTGTCAGGCCGTAGCCCTGGCGGATGCCTGGTAGGTGGA
AGCGTTTGGCCACGGCCTCACCTACCTCCTTGCTGAGCGGCGCCCCGCCGCTGGCGATCTCGTGCAAGTTGCTTAG
GTCGTACTTGTCGATGAGAGTGCTCTTAGCGAAGAAGCTAAATAGTGTGGGCACCAGCAGGGCAGATTGAATCTT
ATAGTCTTGCAAGCTGCGCAAGAATAGCTCCTCCTCGAAGCGGTACATGAGCACGACCCGAAAGCCGCAGATCAA
GTAGCCCAGCGTGGTGAACATGCCGAAGCCGTGGTGAAATGGCACCACGCTGAGGATAGCGGTGTCGGGGATGA
TCTGGTTGCCGAAGATGGGGTCGCGGGCATGACTGAATCGGACACAAGCGGTGCGGTGCGGTAGGGCTACGCCC
TTGGGCAATCCGGTACTGCCACTACTGTTCATGATCAGGGCGATGGTTTTGTCCCGGTCGAAGCTCTCGGGCACGA
AGTCGTACTCGTTGAAGCCGGGTGGCAAATGGGAAGTCACGAAGGTGTACATGCTTTGGAAGCCCTGGTAGTCG
GTCTTGCTATCCATGATGATGATCTTTTGTATGATCGGTAGCTTCTTTTGCACGTTGAGGATCTTTTGCAGCCCTTTC
TTGCTCACGAATACGACGGTGGGCTGGCTGATGCCCATGCTGTTCAGCAGCTCGCGCTCGTTGTAGATGTCGTTAG
CTGGGGCCACAGCCACACCGATGAACAGGGCACCCAACACGGGCATGAAGAACTGCAAGCTATTCTCGCTGCACA
CCACGATCCGATGGTTTGTATTCAGCCCATAGCGCTTCATAGCTTCTGCCAGCCGAACGCTCATCTCGAAGTACTCG
GCGTAGGTAATGTCCACCTCGATATGTGCGTCGGTAAAGGCGATGGTGCCGGGCACCAGGGCGTAGCGCTTCATG
GCTTTGTGCAGCTGCTCGCCGGCGGTCCCGTCTTCGAGTGGGTAGAATGGCGCTGGGCCCTTCTTAATGTTTTTGG
CATCTTCCATGGTGGCTTTACCAACAGTACCGGATTGCCAAGCTTGGCCGCCGAGGCCAGATCCTCTAGAGTCGAG
GAATTCGAAGTTGATGGGCACCTCGCTAGGAAAGGGGGCAGGGCCGGGTTGTCCCCGGCCCTCCCCCCACCCCTG
GGCTCCTGGGATTTGGCACTGCCCGCCTGGCACAGCGGGAGCCCCTCGGCTGGCTCAACTCCTCCGCAGCTAGCC
GCCTTCCCGGCTACCCTGCCTCATTCTCTCACTCCCAGACTTCTTTCCCCGCCCCTGGCTCCCCCTCTGCCTCCGCCCA
CTAGGGCCCCAGCCTTGGGGCCACTGGGACTTGGAGGGGTTAACTAGGAGTGAAGAGTACGGGATCTGCCTTGT
CTTAAAGGAGCCTGGGCGCCTTGGAGAGAGGCTGGGGAGGAGGAAGGAGACCTTGGTGGCTCATGGTGGCTGA
GGCCGATCCTGGGCTGGTGAGTTAGGAGGATGGGCGCTGTGTCCCCCTGAACTCTCAGGGTCTGGTAAACATTCC
TGTAGAATTTCCACTCCTCTCAGCCCAAGACGCCCCCCAGCCCCCCTTTCTGGGCCCCCACCCTCAGCCCAGCCTGC
CAGGCCTCCTCCAATTCCCCTCACACTCGCAGCTTGAACAAGGACTCTCTCTCTCTCTCTCTCTCTCTCTCTCTCTC
TCTCTCTCTCTCTCTCTCTCTCTCTGCATTGACACAGAAGCTGGGACAGGTAGAGACATGCAATGGCATGCTGCCTG
CCCACAGATACCCTCAGATGAGCCGGGTCAGGAGCTTGCCTGGGGAGAAGTCTTACAAATGGAAGGTCACTGCG
GGACTGGCATATGGGGGATCAGGGTACTCACTGAGGGCACATCAACACTCAAACCCACCGAAGCCCACTTGAACA
CAGAAACAGGAATCCAGAGGTGGCAGCAGACTGAAGTGACAACCCCAGCTCTAGCCCAGCCACATGACTTTGGGC

AATGGGCAAATGCCTGTCTTCCTGTACCTCAGTGTGTCTAGCTTTAAGATGGGGACGAGGACAGCCCCTGCTAATG
CAGCAGGCTAGTGCGAGAATGATCTAGGTCAAAGTGCATGCAGTTTCTTGCACAGGGTGGCCCAGATGACGAGG
TGCCCGGGATTTTTGCTACCCATGCTGGTTGTTCCTCTGCCTCAGGATTCTTGTATCCAGCCCTGGCAGATCCGGAG
CCAGTATTAAGCCTCTGCTGCATGTCTGCTTCTATGCCTCTAGGTGTTTGCCATAGAGGGCATGCTCAGATGGCCTC
CTAGTACCCAGTTCTCTTCTTCATTAGTCAGGCTGCTTCTTGATCAAATCATTGAGCTGCTTTTGATCACCACAGAAT
CCCATGTCACCTCCTGGCATCTCTCTCTCTCTCTCCCTTTGCCCTTCTCCATATCCACCACTCCTCCTTCCATCCCCC
TGCCTCCAATCCATCTCTTGGTTGGGATCTAGGAGGATGTTTCTGAGACAGCCCTCCCCCCCATGTCTGTCCTCTAG
ACTCCCCACTTCTCTGATGATCAACTCCAAGCCTCTCAGCTGCTCGCTCTCGGTCCTAACCATAACCACCATCCTGTT
TGTATCTTGATTCAGTGATGTGGCAGCCCCGGCATCTAACCTGCCACCTAGTCTGAGCCCTGGGGTCCTCCTGAAC
GCTCAGTGTGGATTGGTTGCATGGAATCCCAGGGAGAGCTCAGCCTTACAAACCTCTCTCCTATGTGGCTGCCTCT
TTAGAGTCTCACCCTGGTCTAGCCTTGCCCTTTCCCTCCTGGATCCCAGCTATAATTTGCTCCTTCAGTGGCCCGATT
GCTTCTTCCATCACGTTTCCCTTTTAGCAGCCACAGGGTGTTTCTTATCCCACATCCCATCTGATCCTTTCCTTCTCCC
TTCCTTCCCTCTCCGATACCACCTAGCTGTATCCCATCTTCTGCTTCAGCCTTTAGGATAAAGCCACGGGCTTCTTAC
AAGCACCCCTCATTGCCACCTGAGCGTGTCCTCACCTCCCTGGTCAGTTTTCATTTTCTCACCCGAAACAGCTCCCTC
ACAGGCTGTGGAACAGCTTTCTGTGGCACTTTGTCCCAAGCTCCTACACCTCCCTCAGCCCCACCTCTCCTCCCTTTT
CTTTTGCATCCTTTCCAGCCCCCTCCTGTCTAACCTAGCTCTACTCCTTCAATATACGGCTCCCTCTCAGCACCGCCTT
TTTCTGAGATGGTCTCACTATATCACCAAGGGTGGTCTTGAACTCTCAGTCCTACGTGCTTTGCCCTCCAGAGAGTG
CTGGGTGACAGGCAAGCACCACCACACCCAGCTTACCCGGTGCTTTTCATTGGCAGCAGGCCCCGTTTATTGTCAT
TATGGAGTTATTGCACAATTCTGTTTCCTGCCTGTCTCTCCTTTCTGCTGGTGGACGCTGGGAGGACAGCCTGGGG
GATATCGCAGATATTGCTGCAACCCTTAGAAAAGGCCTTGCCATACTGTAGATAAGAAGAAAAAAAGGCATCTCT
GGGGTGAAGTGGAAACTTTAGGGTTCTTTGGAAAGTCGGTTGGATGATATTTTGCTGGGGCAAACACATGAAGG
GACGTTTCATTAAAGCAGACATAGGTGTGAAAGGCTAAGGTAGACTCATGAAGGAATGTTTCGCTGAGAGACACA
CACAGGAGAGAGGATGTTCTGCTAAAGCAAGTGTGTGAAGGGACACGTGATGAAGGATTCTTTGCTAACAGCACT
CAGGTATTGGTCTGCCTTACGTTGCATAGTTGATCTGCTTTTGTTGGGACTCCACAGACTTGGGCTGGCTGCGGCA
GACTTGGGTGGAGGATGCATGATGTTTGGTGGGTGTTGGAGATCGGGCCTAATGCTTTGATTTGACAGGGAATGT
GCTTGTTTGTGCAAACACTAAAGTTAGTTTTTGATTGATCAATAAAGATGCCAGTGACTAATGGCTGGGCAAAGGG
AGGCAGGGTGGGACCCTTAGAGTTGGTGGGGCTAGGACACAGGGGAAAGGAGGAAACGCAGACTTTCAGGATC
ACTGTGAATCACGGGGTTGGGGGGAGCGTGGAGAGAGATGGATCAGATTTAGAGCTGCAGAGGGAACATCATCT
GAAATGTAGGGAAAGCGGCCGCCGGAAAGGCTGCTCAGAAACACCTTGGGCATCAGAGACCAATGGGCCCTACA
GAAGGTAACAGCGCAGCAAAGTTAAAAGTAGATCCAAGGAGGCTGGAGAGGTGGCTCAGCAGTTAAGAGCACT
GGCTGCTCTTCCAGAGGTCCTGAGTTCAATTCCCAGCAACCACATGGTGGCTCACAACCATCTGTAATGGGATCTG
GCGTGTCTGAAGACAGCTACAGTGCACTTACATACATTAAATAAATAAAATCTTTTTTAAGAATGCAAAATATGTAT
TTAATGTTTCTAACCAGTACAAAGACCAAAGGCCACATTTACTCTAGGTTAGTCACAAACCAATTATTTAATAGAAG
CTATAAAATAATTGGGAAGAATATTTGGAATATCTCAAAAGATTTAAAATAGGAGGACACTTTACAAAAATCCGCT
TAGGAAATAAAATTCCTCACCTGTATGTAGAAATGTAACATTGCAGGGGAGCTTATAAAATACAAAGACAAGTATA
AAAGGACTGTGTACTCTAAATCCAAAAACTCAATAATAACCAAATACTTAGTTTTAAAATTTACAGTTCCTGGGTAT
GGACCACATTATTATACACATCTTCCAAGACAGCAATCATTATTCTTCAGTTAACCATGTGAGGTATCGTCTTCTACA
AAATCTTTGGCCATCTCTGCTGTGATCACATCAATGTGACTAACCTTATTTCTGGACTTTACCCCATAGAATTTGTCA
GCTGACTCAAGCAGTTCAGGCCTAAAGGCAGTAGTAATAAACTGGGCATGCACAGCTAGCGCTATAATCATATCT
GACACAGCTTTTCTGTGTTGAGCATCCAAAGCTTGGTCGATCTCATCAAACAGGTAAAAAGGAGCGGGGTCACATT
TCTGAATGGCAAAAATGAGAGCAAGGGCTGCCAGAGATTTCTGTCCTCCTTAAAGCTGTTGCATTTCTCTCAACTCC
CCTTGCTTCCCTGTAAACGACACCCTGACCCCAACTCGTGTGGACTGGTCAACTGTTGGAACACTGCTCTGTGACCC
AGAGCCCCGTTCGCTCTCGCCGCTCCCTTCTCCTTCGTCCTGAGACTGGCTGCCCTCCACATTTCCTTTCTTCATCAC
CAAAGTTGCTTTGCCACCGGGTACCAACTTCTGAAAAACTTCACTGAAGTTCTTAGATACCTGTTTGAAAGTTAACT

GAATAGCTTCATATTTTCTAAGTTCGAGTACATTCATCAATTCCATGATTGAAAGTCTTTTTTTTAAAGTAGATTTAG
AAAGTGTTGAGCCAGGAGTACATGAGGGGAAAGCATGCTCGAGGCTAGCGAGCTCAGGTACCGGCCAGTTAGGC
CAGAGAAATGTTCTGGCACCTGCACTTGCACTGGGGACAGCCTATTTTGCTAGTTTGTTTTGTTTCGTTTTGTTTTG
ATGGAGAGCGTATGTTAGTACTATCGATTCACACAAAAAACCAACACACAGATGTAATGAAAATAAAGATATTTTA
TTGCGGCCTGTCCAATACCTCCCGTACCTTAATATTACTTACTTATCCTTGAGAGACGTACTAGTAACCCTGATAAAT
GCTTCAATAATATTGAAAAAGGACGAGTATGAGCATCCAACATTTTCGTGTCGCACTCATTCCCTTCTTTGCGGCAT
TTTGCTTGCCTGTTTTTGCACACCCCGAAACGCTGGTGAAAGTAAAAGATGCTGAAGATCAACTGGGTGCAAGAGT
GGGCTATATCGAACTGGATCTCAATAGCGGCAAGATCCTTGAGTCTTTCCGCCCCGAAGAACGATTCCCGATGATG
AGCACTTTCAAAGTACTGCTATGTGGCGCGGTGTTGTCCCGTATAGACGCCGGGCAAGAGCAGCTTGGTCGCCGT
ATACACTACTCACAAAACGACTTGGTTGAGTACTCGCCGGTCACGGAAAAGCATCTTACGGATGGCATGACGGTA
AGAGAATTGTGTAGTGCTGCCATTACCATGAGCGACAACACCGCGGCCAACTTACTTCTGACAACGATCGGAGGC
CCTAAGGAGCTGACTGCATTTCTTCATAATATGGGTGATCATGTGACCCGGCTTGACCGCTGGGAACCAGAGTTGA
ACGAAGCCATACCGAACGACGAGCGTGATACCACGATGCCAGTAGCAATGGCCACAACTCTTCGGAAACTACTCA
CTGGCGAACTTCTTACTCTAGCATCACGACAGCAGCTCATAGACTGGATGGAGGCGGACAAAGTAGCAGGACCAC
TTCTTCGCTCGGCCCTCCCTGCTGGCTGGTTCATTGCTGACAAATCGGGGGCCGGTGAACGCGGCTCTCGCGGCAT
CATTGCTGCGCTGGGGCCTGATGGTAAGCCCTCACGAATCGTAGTGATCTACACGACGGGGAGTCAGGCCACTAT
GGACGAACGAAATAGGCAGATCGCTGAGATCGGTGCCTCACTGATCAAGCACTGGTAACCACTGCAGTGGTTTAG
CATTTGCGGCCGCTGTCAGACCAAGTTTACTCATATATACTTTAGATTGATTTAAAACTTCATTTTTAATTTAAAAGG
ATCTAGGTGAAGATCCTTTTTGATAATCTCATGACCAAAATCCCTTAACGTGAGTTTTCGTTCCACTGAGCGTCAGA
CCCCGTAGAAAAGATCAAAGGATCTTCTTGAGATCCTTTTTTTCTGCGCGTAATCTGCTGCTTGCAAACAAAAAAAC
CACCGCTACCAGCGGTGGTTTGTTTGCCGGATCAAGAGCTACCAACTCTTTTTCCGAAGGTAACTGGCTTCAGCAG
AGCGCAGATACCAAATACTGTTCTTCTAGTGTAGCCGTAGTTAGGCCACCACTTCAAGAACTCTGTAGCACCGCCT
ACATACCTCGCTCTGCTAATCCTGTTACCAGTGGCTGCTGCCAGTGGCGATAAGTCGTGTCTTACCGG

** Note: For a fully finished plasmid, the FASTA file header will say "CIRCLE". As result of our final assembly QC, the first 52 basepairs of the sequence are repeated at the end of the sequence. Prior to importing the complete plasmid sequence into another sequence analysis program, please remove the last 52 basepairs at the end of the sequence.
